# The validity of androgen assays

**DOI:** 10.1080/13685530701483738

**Published:** 2007-08-17

**Authors:** Malcolm Carruthers, Tom R. Trinick, Michael J. Wheeler

**Affiliations:** 1 Centre for Men's Health, London, UK; 2 Department of Chemical Pathology, The Ulster Hospital, Belfast, UK; 3 Department of Chemical Pathology, St. Thomas' Hospital, London, UK

**Keywords:** Review, androgen assays, validity, testosterone, distribution, interpretation, resistance

## Abstract

Problems in the measurement of androgens and in interpreting results have been reviewed and classified as follows:

**Preanalytical factors:**

The exact sampling conditions in relation to circadian and seasonal variations, diet, alcohol, physical activity and posture.

**Physiological and medical factors:**

Androgen levels vary according to the patient's general health, stress, sexual activity and smoking habits.

**Analytical variables:**

Sample preservation and storage variables are often unknown.

The different androgen assays used have widely differing accuracy and precision and are subject to large inter-laboratory variation, which especially in women and children can render the results of routinely available direct immunoassays meaningless.

**Interpretation of results:**

Laboratory reference ranges vary widely, largely independent of methodology, and fail to take into account the log-normal distribution of androgen values, causing errors in clinical diagnosis and treatment. Other unknowns are antagonists such as SHBG, estrogens, catecholamines, cortisol, and anti-androgens. As well as age, androgen receptor polymorphisms play a major role in regulating androgen levels and resistance to their action.

**Conclusions:**

Though laboratory assays can support a diagnosis of androgen deficiency in men, they should not be used to exclude it. It is suggested that there needs to be greater reliance on the history and clinical features, together with careful evaluation of the symptomatology, and where necessary a therapeutic trial of androgen treatment given.

## Introduction

Androgen deficiency has been implicated as an important contributory factor in coronary heart disease [1], metabolic syndrome and diabetes in men [2], desire disorders in women [3], and mental [4] and physical [5] aging in both sexes. It is of increasing clinical importance, therefore, to assess the validity of androgen assays and their interpretation [6]. This will be considered sequentially from taking a sample and analysing it, to interpreting the result.

## Pre-analytical factors

### Circadian variation

About 50–60% of the total testosterone (TT) is bound strongly to sex hormone binding globulin (SHBG). A further 40–50% is weakly bound to albumin, which together with the 1–3% free testosterone (FT) makes up the so-called ‘bioavailable’ testosterone (BT). Therefore, the combination of increased testosterone synthesis at night, with decreases in its binding proteins due to the haemodilution of recumbency, causes more marked circadian variation in BT (57%) and FT (68%) than in TT (45%) [7]. The reduction in circadian variation in TT and physical activity with ageing may however reduce this effect.

### Seasonal variations

Circannual variations of 19% in TT and 31% in FT were described by Svartberg et al. [8], who found lowest levels in summer, with a peak in the autumn, with similar but variable results reported in other studies according to geographic location.

### Diet

One of the important variables, which is often not reported in studies of reference populations and patients to establish androgen deficiency, is whether samples have been taken in the fasting state, or after low or high glycaemic meals, both of which might fall within attempts to define them as ‘a light breakfast’ [9]. It has been known since 1973 that testosterone levels in normal males can fall in response to oral glucose by over 30%, depending on age, time and glucose load [10]. Recently it has been found that a standard 75 g oral glucose load in younger men resulted in a 15% reduction of fasting TT levels after 30 minutes, which continued for up to 3 hours [11]. This was shown to be due to an increase in glucagon-like peptide-1 (GLP-1) reducing the pulsatile release of testosterone, and that the effect was independent of changes in LH.

The most important long-term nutritional effects appear to be mainly on SHBG, which is decreased by high protein, high fat diets such as Atkins, and increased by vegetarian and high fibre diets [6]. These changes may be largely via insulin levels, which tend to be lower in vegetarians, and are inversely related to SHBG. Also, high levels of free fatty acids interfere with the binding of sex steroids to SHBG, which can affect the level of FT [12].

### Alcohol

Alcohol in low doses has been shown to raise testosterone levels by 19% in men [13] and women [14]. Conversely, acute alcohol intoxication, especially if accompanied by strenuous exercise, can reduce testosterone levels for up to 22 hours by 23% [15]. Long-term excess alcohol can cause irreversible damage both the Leydig and Sertoli cells in the testes, contribute to obesity, raise oestrogen levels, and cause sustained androgen deficiency.

### Physical activity

Besides changes due to haemoconcentration, depending on age and the fitness of the individual, various intensities of different forms of exercise can cause wide variations in androgen levels [16]. This can result in the paradox of young endurance athletes having subnormal TT and FT [17].

## Physiological and medical factors

### Illness

Reduced androgen levels have been reported in serious illnesses, ranging from severe trauma, and coronary heart disease to liver disease, though it is always difficult to establish which came first. The MMAS study showed that as a group the 18% apparently healthy men in their follow-up study had TT levels 15% above the rest of the 1,156 men remaining out of the original group of 1,709 [18].

### Stress

Both excessive and unpleasant physical and mental stress can activate the hypothalamo-pituitary-adrenal axis and reduce either the amount or activity of androgens. Christiansen in 2004 [16] reviewed the effects of various types of stress on testosterone secretion that included a variety of mental and physical stressors.

### Sexual activity

Serum and salivary free testosterone have been shown to increase in both men and women with a wide variety of sexual activity, including masturbation [16].

### Smoking

Smokers have been found to have both total and free testosterone levels 5–15% higher than non-smokers [19]. Increases in both TT (9%) and SHBG (8%) were found by Field et al. [20], while DHT increased 14%. The immediate effects of nicotine do not appear to have been studied, and so it is unclear what the effect of smoking on the morning of the test might be.

## Analytical variables

### Type of sample, separation and storage

Recent studies have shown that samples for testosterone measurement should be separated within 6 hours at room temperature, or can be stored at plus 4°C for up to 48 hours before separation [21]. When frozen at minus 20°C, they are stable for up to three months, and for over six months at minus 70°C. In some epidemiological studies, these limits have been carried to extremes, and samples re-analysed after periods up to 10 years [18], though changes in methodology gave results over 80% higher for TT and therefore FT in the later analyses, making the stability of the samples uncertain. Certainly, it is inadvisable to repeatedly thaw and freeze samples, as proteins such as SHBG are likely to denature, giving different results for BT and CFT.

Although the use of a serum or plasma sample was acceptable for extraction assays of testosterone, they are not both valid for use in direct assays. For example the Bayer Centaur assay is validated for serum only, as is the Immulite Analyzer. With the latter values for TT obtained with heparinized plasma are reported as 10% lower [22] and SHBG 3–6% lower [12]. EDTA and citrated plasma samples give grossly different results for all direct assays and should never be used.

Plastic blood collection tubes are now used extensively and have been compared with glass collection tubes in which clot retraction is more rapid and complete. Direct comparison however has shown no significant difference in testosterone levels between samples collected in glass and plastic tubes [23].

Even sample tube surfactant has been reported to cause a positive bias in TT estimations, up to +28% for two of the methods in common use [24]. The same NHS Medical Devices Agency (MDA) Alert reported biases on FSH of −43%, total T3 of +58%, total T4 of +34%, and B12 and folate up to +84%.

The immediate and impractical action required of the laboratories receiving the alert was to ‘consider advising clinicians who have commissioned these tests on the need to recall or retest patients whose test results may have been affected’.

However in a later study [25] of 15 immunoassays performed on Bayer Advia Centaur using blood specimens collected into four different BD Vacutainer tubes (plain, old and newly released BD SSTII Advance, and BD PSTII), the plain tubes and old SSTII Advance tube results showed no bias for testosterone, CA15-3, follicle-stimulating hormone and folate assays, but gave a positive bias for cortisol and a negative bias for vitamin B12.

Compared with plain tubes, BD PSTII tubes gave no significant bias for thyroid function tests, prolactin, parathyroid hormone, and CA125, but gave a negative bias for steroid assays, and a positive bias for gonadotrophins. The results obtained using new BD SSTII Advance tubes were generally comparable with those of plain tubes, but only for cortisol did this study support the bias described by the MDA. They conclude that BD PSTII tubes should not be used for steroid hormone measurements on the Bayer Advia Centaur instrument.

### Methodology

Commercial direct automated assays have mostly replaced the old extraction assays conferring advantages of ease of analysis, speed and throughput. However there has been severe criticism of the analytical accuracy of assays in the measurement of testosterone in female serum [26]. The functional sensitivity of the older extraction assays was 0.1 nmol/L (3 ng/dl) whilst direct assays are unreliable below 1.0 nmol/L (30 ng/dl) [21]. In addition some commercial assays show intra-assay imprecision in female sera ranging from 8.9–21.3% whilst the figure for males was an acceptable 3.3–5.5% [27]. Variations in accuracy between testosterone methods and a GCMS standard have been shown to be up to 218% in America [28] and 96% in Australia [9]. Even between laboratories using the same methods, and between batches of reagents within American laboratories, these are up to 23%, and only 60% of TT levels within the adult male range were within 20% of target quality control values [28].

Within laboratory coefficient of variation, especially at low levels of testosterone and SHBG can be as high as 16% [22] and 10% respectively, errors which are compounded in the calculation of BT and FT.

Another recent paper lends further support to the view that immunoassay is unsatisfactory for measuring the testosterone concentrations typically found in women and children, and that bench-top tandem mass spectrometers are a desirable alternative technology for in such cases for measurements in the clinical laboratory despite the additional cost [29].

This study used stable-isotope dilution liquid chromatography-tandem mass spectrometry (ID/LCMS/MS) to measure testosterone in plasma and serum. Intra- and interassay imprecision was <15% in the range 0.3–49 nmol/L. Recovery of testosterone added to samples at concentrations of 0.625–20 nmol/L was 96% (CV = 12%; n = 26). Correlation with isotope-dilution gas chromatography-mass spectrometry for 20 pools of clinical samples (range, 0.5–38.5 nmol/L) was 0.99. Various steroids added to double charcoal-stripped serum showed no interference at the retention time of the testosterone peak. It was concluded that ID/LC-MS/MS has improved accuracy compared with immunoassay and the low sample volume and simplicity, rapidity, and robustness of the method make it suitable for use as a high-throughput assay in routine clinical biochemistry laboratories.

Similarly, a reference measurement procedure has also been described to measure FT using isotope dilution mass spectrometry following ultrafiltration [30]. The method gave maximum within-day, between-day, and total CVs of 3.0%, 3.1%, and 4.3%, and satisfactory correlation with indirect equilibrium dialysis and symmetric dialysis. However, they also demonstrated that ‘a degree of discordance remains, which may require a decision from an authoritative organization on the recommended procedure to measure free hormone concentrations’.

More recently this group has reported the comparison of four routine analog assays for serum free testosterone, and the calculated free testosterone (CFT) with the previous reference measurement procedure [31]. While the CFT was in good agreement with the reference method there were substantial differences in analytical quality of the analog FT assays. The results suggested that after extending the validation with a larger variety of samples, recalibration of some analog assays might be worth further investigation.

A recent study of the accuracy of 10 immunoassay methods compared with the reference isotope dilution gas chromatography-mass spectrometry method (ID/GC-MS) showed that mean immunoassay results in women were 46% higher than ID/GC-MS whilst the mean results in men were 12% lower [32]. The authors concluded, along with many others, that ‘None of the immunoassays tested was sufficiently reliable for the investigation of sera from children and women, in whom very low, e.g. 0.17 nmol/L (5 ng/dl), and low, e.g. <1.7 nmol/L (50 ng/dl), testosterone concentrations are expected’.

Recent data from UKNEQAS showed that whilst mean recovery was 99.6% in male serum, the mean recovery in female serum was 70.5% for the major immunoassay methods [33]. The concentration of SHBG has been shown to significantly affect the testosterone result and agreement between methods [27].

The association constant of SHBG, on which the calculation of BT and CFT depends, has been reported by various authors as being between 0.6 and 1.9 × 10^9^ l/mol [12]. Changing from higher to lower values of this constant can increase calculated BT by 123% and FT by 254%.

Many of the above problems are well summarized in a recent position statement by the Endocrine Society reviewing evidence from published sources, the College of American Pathologists, and the clinical and laboratory experiences of the five very experienced authors [34]. They emphasize that the TT concentrations in blood vary over three orders of magnitude depending on age, gender and the presence of disease, and that other steroids of similar structure and abundance in the circulation lead to assay interference. There is a lack of age and gender-related normal ranges using standardized assays, and little agreement on whether TT or the small amount of FT is the most useful clinical measure.

Following a detailed review of methods for measuring TT and FT, listing strengths and shortcomings of each, and discussion of the problems and clinical utility of testosterone measurement in the different ages and sexes, they give suggested normal ranges only for adult males. Their authoritative conclusion is that ‘This review demonstrates that the manner in which most assays for TT and FT are currently performed is decidedly unsatisfactory’.

## Interpretation of results

### Reference ranges

A key study emphasizing how the choice of a reference range for assays can totally alter the diagnostic criteria for androgen deficiency was a review of the ranges used in 25 laboratories in Eastern America [35]. Twelve were leading academic laboratories, 12 community medical laboratories and one a national laboratory, the largest in the USA. All of the academic labs, and eight of the community centres performed TT measurement, using eight different methods between them. FT estimations were performed by six of the academic labs, but only one of the community labs, using four different methods, with SHBG being available in five, but BT and CFT were only quoted in two of these. FT by equilibrium dialysis was only offered by the national laboratory ‘only upon special request’.

Of the 25 labs, there were 17 and 13 different sets of reference values for TT and FT respectively. Apparently independent of methodology, the low reference value for TT ranged from 130 to 450 ng/dL (4.5–15.6 nmol/L – 350% difference), and the upper from 486 to 1,593 ng/dL (16.9–55.3 nmol/L – 325% difference). For FT the lower values varied between 5.0–13.5 pg/ml (174–468 pmol/L – 270% difference) and the upper 19–54.7 pg/ml (660–1,896 pmol/L – 290% difference).

No laboratory performed independent valuation of the manufacturer's reference values, which were based on standard Gaussian distribution analysis of largely unpublished data, using 2.5% and 97.5% cutoff points regardless of any clinical correlation.

As this article points out, if up to a third of men over the age of 50 are clinically androgen deficient, as suggested by for example by Heinemann's Aging Male Symptoms scale [36], then using these widely varying reference ranges obtained with different methodologies, and an inappropriate statistical model, will exclude a large majority of men who might benefit from testosterone treatment. This is made more confusing by the age-related normal ranges reported by four of the 12 academic centres for TT, and seven of the total for FT.

Of the small proportion who are treated, with an inappropriately low upper range, ‘clinicians may become unnecessarily concerned that therapeutic doses of testosterone treatment are excessive if TT or FT results are higher than reference values’.

Given the choice, 92% of lab directors indicated that clinically relevant threshold values would be preferable to current reference values, but would look to national panels or speciality societies for these. How such authorities could allow for different clinical indices of suspicion and laboratory methodologies is unclear.

This article concludes that ‘these results indicate that the current use of testosterone reference values is confusing and inadequate. There is a clear need for standardization and more clinically relevant reference values to guide clinicians in the diagnosis and treatment of hypogonadism.’

### Age

The data from the Massachusetts Male Aging Study (MMAS) of 1,709 men aged 40–70 has confirmed the longitudinal trend for TT to decrease by 1.6%, BT by 2.5%, and FT by 2.8% per annum, the greater fall in the latter being due to rising SHBG levels. Over the 30 year age range studied, this amounts to a drop of 48% in TT, 75% in BT, and 84% in FT. This raises the question of whether lower levels of these fractions found in older men should be accepted as ‘normal’, and go untreated, even if associated with symptoms of androgen deficiency and its related disorders.

### Log-normal distribution of hormones

Observations in ‘normal subjects’ have shown that androgen values are log-normally distributed [37], usually revealed by the fact that the distribution covers more than a two-fold range, as in the case of most hormones.

In spite of this, ‘improper statistics’ [38] have continued to be used to characterize the normal range of biochemical and endocrine measurements.

## Application of log transformation to ‘reference ranges’

Population studies have consistently shown that testosterone data is skewed and that log transformation of the data should be performed [9,18]. A significantly different picture of conditions associated with androgen deficiency in the adult male is likely to result from this simple but important statistical change.

**Figure 1:**
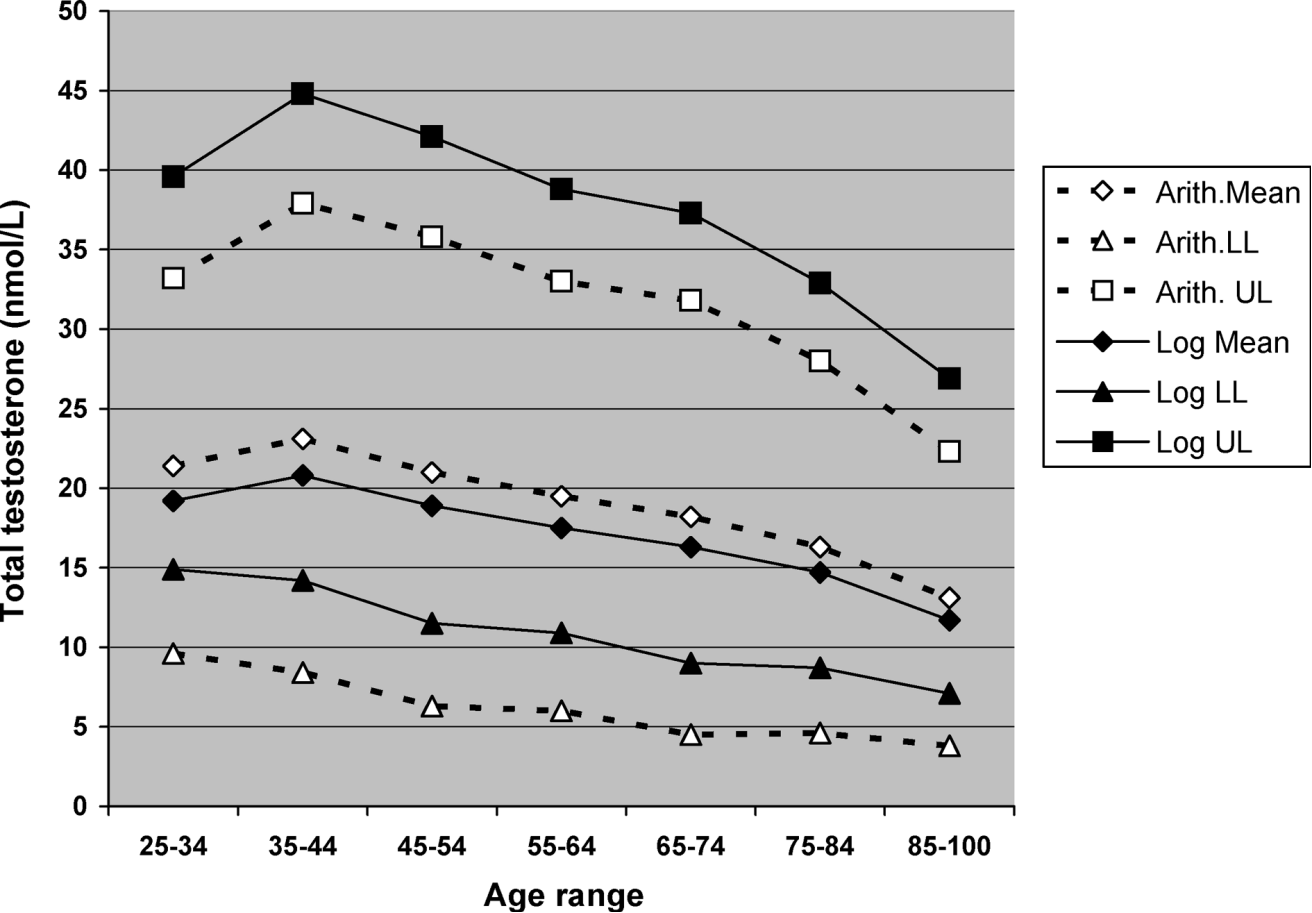
Changes in means and 2 SD ‘reference ranges’ for total testosterone levels at different ages according to the figures of Vermeulen [39] for 249 healthy men, comparing figures calculated from arithmetic and logarithmic distributions [6].

The differences in the range of androgen values given by the arithmetic and logarithmic distributions can totally alter reference ranges. For example, a study of the distribution of androgens in patients with symptoms of androgen deficiency [6] showed that on average, the logarithmic transformed means are 9% lower than the arithmetic means, and the lower and upper limits of the logarithmic distribution increased by 30% of the arithmetic means. Applying these log conversion values to the study of 249 ‘healthy’ men by Vermeulen [39] gives the results seen in Figure 1.

Though it needs to be confirmed by re-analysis of the original investigators data, and results from an older patient population may be more skewed than those from those in young and healthy men, for example the approximately 10% increase in upper and lower limits seen in one such study [9], it may be possible to estimate more appropriate ‘reference ranges’ from the figures given in the existing literature.

The clinical importance of these two key factors, analytical variation and the log transformation of data used in establishing ‘reference ranges’, is clearly demonstrated by this recent study [9]. Using a reference panel of sera from healthy eugonadal young men with verified normal reproductive function, major differences were found to exist between commercial TT immunoassays, as well as divergence from the GC/MS standard. The authors concluded ‘This impairs their clinical diagnostic utility and requires substantial improvements in automated T immunoassay technologies or a switch to GC/MS methods’.

**Figure 2:**
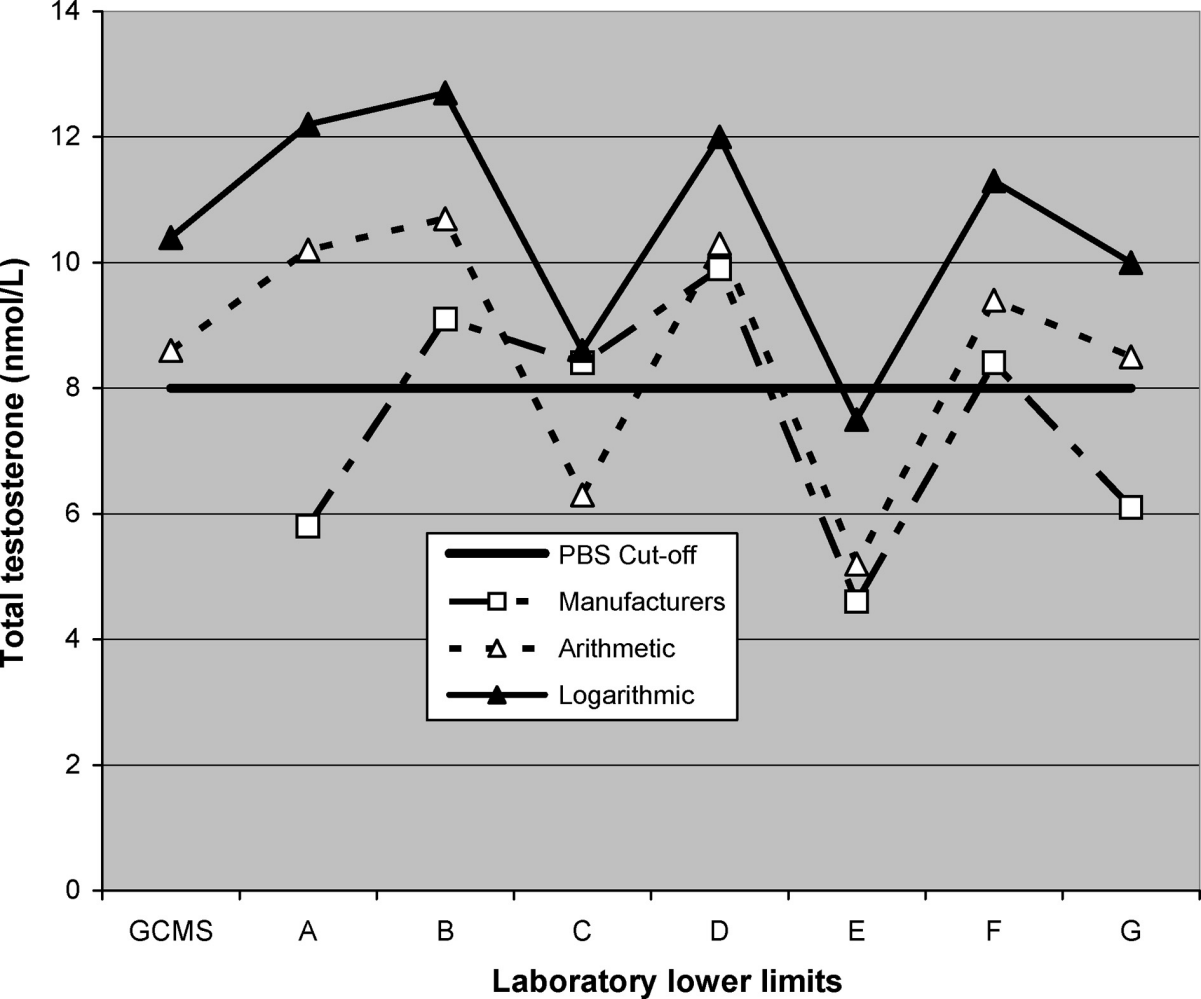
Lower Limits of TT in relation to PBS ‘cut-off limit’ based on results from 7 different ‘leading Australian laboratories’ (A–G) using various fully automated methods for measuring TT [9] compared to the GC/MS standard reference method. The lines connect the limits in each lab derived from arithmetic and logarithmic calculations, compared to the manufacturers quoted reference range.

As an example of the direct clinical relevance of these factors, when combined with differences in mathematical calculations of the lower end of reference ranges, this study appeared to totally invalidate the criteria for Australian men to qualify for testosterone treatment under the Pharmaceutical Benefit System (PBS) [40], which sets a limit of 8 nmol/L, (230 ng/dl) for the diagnosis of ‘hypogonadism’ (Figure 2).

These figures suggest that the majority of men falling within 4 nmol/L (115 ng/dl) of the PBS range would fail to qualify for testosterone treatment under the present regulations.

The frequently urged solutions to switch to mass spectrometry-based methods, or for each laboratory to establish its own reference range from a large pool of healthy, reproductively normal young men, is seldom practical outside research centres, and it is clinically undesirable to have local variations in methodology affecting the interpretation of key endocrine results.

## Ethnic differences

It has recently become apparent that genetic differences can affect the level of androgens, and SHBG in different races, requiring different interpretation of these figures in relation to ethnic mix. A study in Manchester [41] showed that mean TT levels in Pakistani men were 28% lower than Europeans, and 23% lower than those of African-Caribbean origin. The corresponding means for FT were 24% and 25% lower.

Racial, familial and individual variations in the androgen receptor can affect the level of both androgens and gonadotrophins, as well as organ-specific sensitivity or resistance to their actions, and therefore the resulting symptoms and signs of androgen deficiency [42–44].

## Choice of androgen measures in the diagnosis of androgen deficiency

As can be seen from the above discussion, there is no perfect measure of androgen activity, though some appear more useful than others.

### Total testosterone (TT)

Though unfortunately TT is the most commonly measured and quoted, it is a poor indicator of clinical androgen activity, falling least with age, and having the weakest relationship with most clinical states and their response to testosterone treatment.

These factors underline the view of Atkinson et al. [45] that ‘Because of the variability in serum testosterone concentration in the normal male during the day, from person to person, and among assays, there is no accepted testosterone value used as a cut-off to define testosterone deficiency. Symptoms, etiology, clinical impression, and a very low or low-normal testosterone aid the diagnosis of hypogonadism’.

### Free androgen index (FAI)

This has the merit of being easy to calculate, and makes some allowance for the important effect of SHBG. It has however been attacked on theoretical grounds by Kapoor et al. [46] when used in men, on the basis that the binding capacity of SHBG needs to greatly exceed the concentration of its ligand testosterone for the equation to be valid. Vermeulen et al. have also shown it to correlate poorly with FT results obtained by equilibrium dialysis [47].

### Bioavailable testosterone (BT)

Also known as ‘free and weakly-bound’ testosterone, this has the advantage that the ammonium sulphate precipitation is simple, cheap and direct, and has been recommended for screening for androgen deficiency [48].

Though popular in America and Canada, it is however seldom measured in Europe, and obscures the information gained by measuring SHBG. There is also the question of whether the testosterone, weakly bound to albumin, is actually free and biologically active in its short transit through the capillaries in all parts of the body, e.g. the brain. The albumin-bound fraction is also considerably greater, but less variable, than the free fraction, and variations in the former may mask smaller but potentially more important changes in the latter, which is usually only 5–10% of the Bio-T.

### Calculated free testosterone (CFT)

As it has been found that CFT and FT measured by equilibrium dialysis [47] show a higher correlation than any of the other measures, it was concluded that ‘calculated FT is a reliable index of FT, that calculated non-specifically bound T reflects non-SHBG-T, and that immuno-assayable SHBG is a reliable measure of SHBG binding sites’. These key statements from this detailed study must be regarded as the definitive ideas at present in this complex field, and make CFT the laboratory measure of choice. A nomogram for deriving CFT from total testosterone and SHBG, using the equation provided by Vermeulen et al. [47], has recently become available [6].

### Salivary testosterone (ST)

In research studies where frequent sampling is required, especially biosocial and population studies to detect androgen deficient states, salivary testoster-ones are potentially very useful. However, they require careful collection and preservation in special plastic sampling devices under standardized conditions, without blood contamination, and the use of suitably sensitive assay methods to avoid ‘The trouble with salivary testosterone’ [49].

Goncharov et al. showed they are stable for up to five days at room temperature, enabling samples to be transported by post, and for up to six months at −20°C [50]. Development of ultrasensitive luminescent assays, which the authors recommend as being more sensitive and therefore preferable to radio-immunoassay methods for this purpose, has made it possible to measure the testosterone in the saliva of normal and androgen deficient men, with a mean precision of 3.5%, 4.7% and 7.8% for intra-assay, inter-asssay and between-lot variation. This study also showed that morning ST levels correlated well with CFT in both groups.

Another study looking at age-related change in salivary testosterone among Japanese males used a radioimmunoassay kit modified for saliva [51]. There was a significant decrease in salivary testosterone values from 20s to 40s and older but no further decline after 40 through 90 years old. These results suggest that neither a constant decrease of salivary testosterone values or markedly reduced intraindividual fluctuations are universal aspects of aging. Older males may maintain relatively high testosterone levels compared to younger men and a relatively ‘robust’ neuroendocrinological system. The findings may however be related to the low levels of obesity, and exceptional longevity of Japanese males [52].

They are certainly inconsistent with the findings of Morley et al. [53] who looked at salivary testosterone in 1,454 American men between the ages of 20 to 89 years and showed a 47% decline across that age range. They also showed that salivary testosterone was highly correlated with BT, CFT and TT. The ST levels related significantly to symptoms of androgen deficiency on both the Saint Louis University ADAM questionnaire and the Aging Male Symptom scale. However, it was concluded that ‘Salivary testosterone is not a better assay than other measures to diagnose hypogonadism’.

## Conclusions

Many of the factors discussed in relation to the validity of catecholamine assays [54] nearly 40 years ago seem equally applicable to the current state of androgen measurement and interpretation, but have potentially more serious clinical consequences.

There appear to be so many confounding variables in obtaining, preserving and analysing androgen samples (Table I) that conventional interpretation in relation to arbitrary reference ranges seems inappropriate. It is suggested that in men, laboratory assays can support, but not always exclude, a diagnosis of androgen deficiency. A typical symptoms as assessed by a complete physical examination and fully validated questionnaires such as the Aging Male Symptom Scale [36] may in cases of doubt indicate a therapeutic trial of testosterone treatment in patients without contraindications.

**Table I:** Summary of factors affecting androgen assays in men, showing maximum reported variations in total testosterone (TT) and free testosterone (FT) due to each factor expressed as a percentage. NB: For women, the analytical variations are greater, and other than the age-related drop with menopause, the influence of the other factors is largely unknown.

Factor	TT%	FT%
*Sampling*		
Circadian [7]	45	68
Seasonal [8]	19	31
Diet [6,10–12]	30	30
Alcohol [13,15]	42	42
Physical activity [6,16,17]	50	50
*Medical*		
Illness [6,18]	15	15
Stress [6,16]	80	80
Sexual activity [16]	10	10
Smoking [19,20]	15	15
*Analytical*		
Serum v plasma [12,22]	10	6
Separation, storage [24]	28	28
Between methods [9,28]	218	218
Between labs and kits [28]	23	23
Coefficient of variation [22]	16	25
Association constants [12]	–	254
*Interpretation*		
Reference values [18,35]	350	290
Log distribution [6,9]	30	30
Ethnic differences [41]	28	25

In women and children, unless GC/MS is used, methodological inaccuracies alone can invalidate testosterone measurements using the commonly available direct immuno-assays.
